# Supplementary material for: Unsupervised deep learning identifies semantic disentanglement in single inferotemporal face patch neurons
Source: Nat Commun. 2021 Nov 9;12:6456. doi: 10.1038/s41467-021-26751-5 (PMC8578601; doi:10.1038/s41467-021-26751-5)
Supplement: Supplementary file 1 — Supplementary information. [file 41467_2021_26751_MOESM1_ESM.pdf]

Unsupervised deep learning identifies semantic disentanglement in single inferotemporal face patch neurons

Irina Higgins[1†*], Le Chang[2,3†], Victoria Langston[1], Demis Hassabis[1,4]
Christopher Summerfield[1,5], Doris Tsao[2,6], Matthew Botvinick[1,4]

[1]DeepMind, London, UK, [2]Caltech, Pasadena, USA
[3]Chinese Academy of Sciences, Shanghai, China
[4]University College London, London, UK, [5]University of Oxford, Oxford, UK
[6]Howard Hughes Medical Institute, Pasadena, USA

*To whom correspondence should be addressed; E-mail: irinah@google.com
†These authors contributed equally

Supplementary Information

Supplementary Note: Performance on heldout data.

To ensure that the models do not overfit to the 2,100 faces during training, all models (apart from VGG) were trained on the same set of faces, which were mirror flipped with respect to the images presented to the primates. Supplementary Figure 4a shows that this indeed resulted in a significant shift between the train and test data distributions. This figure demonstrates that to three autoencoding models – AE, VAE and β-VAE – mirror flipped versions of the images used for training are as different from the originals used for testing as completely novel heldout faces coming from the same data distribution (statistically significant difference between train and test, and train and heldout reconstruction accuracy, all $p < 0.01$, two-sided Welsch's t-test; no statistically significant difference between test and heldout reconstruction accuracy, all $p > 0.05$, two-sided Welsch's t-test). This suggests that the results presented in Figs. 3-5 should hold more generally to any held out data coming from the same data distribution.

To validate whether this is the case further, we tried to repeat the alignment score calculations used to produce the results shown in Figs. 3b and 4a using the novel (heldout) 62 faces that the models never saw during training. Due to the small number of samples, however, we struggled to perform the Lasso regression step necessary for calculating the alignment score – all models struggled to explain the variance in the noisy neural data. Indeed, when we repeated the steps exactly, we found that on average across all models the variance of 84.8% of 159 neurons was

not explained at all ($R^2 = 0$), this is compared to 1.32% of neurons when using 2,100 faces. Hence, we used a modified pipeline for the Lasso regression steps. Instead of splitting the data into train and test sets, and using training data for 10-fold cross-validated Lasso regression and test data for calculating variance explained (used in further model selection), we used all 62 data points for both training and testing. Furthermore, instead of using the α that produces the most sparse weight matrix with MSE within one standard error of the minimum achieved across all values of α considered, we instead selected the α that produced the lowest MSE. These changes lowered the proportion of neurons whose response variance we could not explain down to 35.8%, albeit to the potential detriment to the quality of the learnt mapping that underlies the alignment score calculations, hence rendering the alignment scores less reliable. Supplementary Figure 4b demonstrates that the resulting alignment scores are broadly similar to those calculated on 2,100 faces shown in Figs. 3b and 4a. The only major difference is that VGG (raw) results appear comparable to those of β-VAE. We believe, however, that this is likely an artefact caused by the noise inherent to the low data regime.

We further verified the main results presented in the paper by calculating the average correlation ratio and average unit proportion scores using the novel 62 heldout faces. Both scores are based on correlations between the responses of single neurons and single model units. Once again we see that the low signal to noise ratio of neural recordings results in significantly noisier calculations in the low data heldout regime (Supplementary Figure 5a), which in turn affects the resulting average correlation ratio (Supplementary Figure 5b) and in particular average unit proportion (Supplementary Figure 5c) scores. The broad message of the results from the main paper, however, still holds: β-VAE has significantly higher ratio between the maximum correlation and the sum of such correlations between neural firing responses and latent unit activations; there is also more heterogeneity among β-VAE units that achieved maximum correlation with the neural responses than among the units for other models (although this measure is particularly prone to noise in correlation calculations). Together, the results in Supplementary Figure 5b,c validate the alignment results from Supplementary Figure 4b. Note that according to the average correlation ratio and average unit proportion measures, VGG (raw) scores significantly lower than β-VAE and even several other baselines, thus serving as further evidence that the non-significant alignment results from Supplementary Figure 4b may be due to noise.

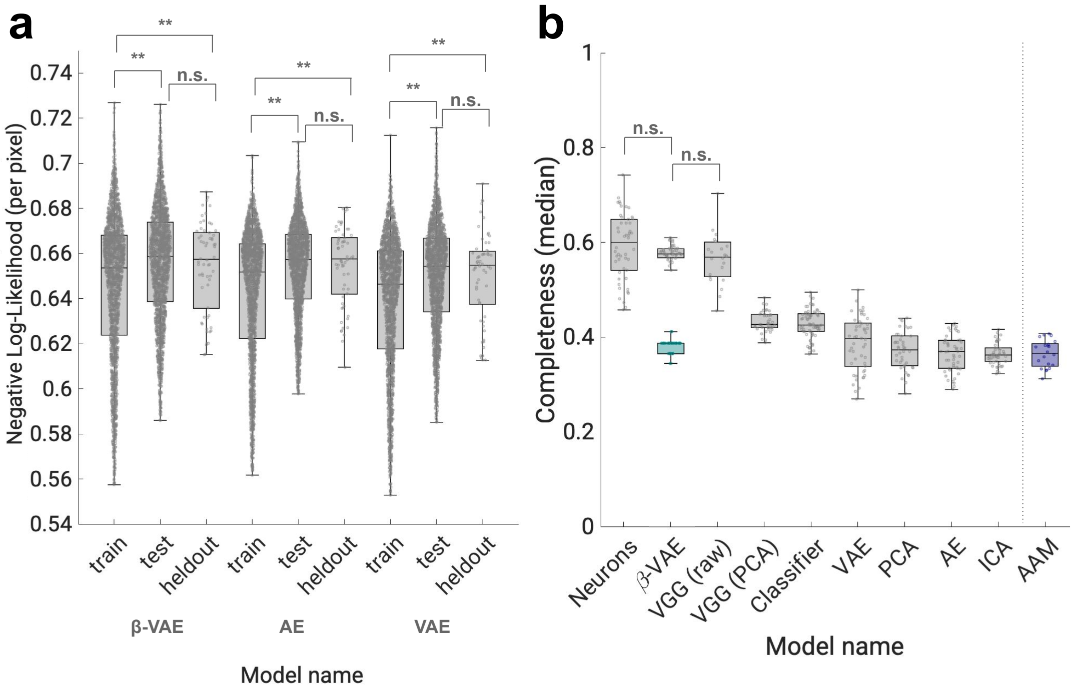

Supplementary Figure 1: **Alignment results broadly generalise to heldout faces. a.** Circles, Negative Log Likelihood (NLL) averaged per pixel for 2,100 face images under the learnt model distribution. Lower is better, with the perfect model scoring 0. Results are presented for the model instances shown in Figure 6c. Train, same mirror-flipped 2,100 faces used for training the models. Test, original 2,100 faces that were presented to the primates. Heldout, novel 62 faces not seen during training. No significant difference is found between test and heldout NLL results (all $p > 0.05$; β-VAE $p = 0.6461$, AE $p = 0.9127$, VAE $p = 0.4269$ two-sided Welsch's t-test); train and test (all $p < 0.01$; β-VAE $p = 0.2359e - 31$, AE $p = 0.0$, VAE $p = 0.0$, two-sided Welsch's t-test), as well as train and heldout NLL results are significantly different across all models (all $p < 0.01$; β-VAE $p = 0.2359e - 3$, AE $p = 0.0063e - 3$, VAE $p = 0.0001e - 3$, two-sided Welsch's t-test). β-VAE train NLL results are significantly higher than AE or VAE train NLL results (all $p < 0.01$, two-sided Welsch's t-test). Boxplot center is median, box extends to 25th and 75th percentiles, whiskers extend to the most extreme data that are not considered outliers, outliers are plotted individually. Source data are provided as a Source Data file. **b.** Alignment scores calculated over 62 heldout faces. No significant difference is found between the alignment scores of neuron subsets, β-VAE and VGG (raw) (all $p > 0.01$, β-VAE $p = 0.045$, VGG (raw) $p = 0.2191$), while the other models are statistically significantly different (all p¡0.01; Classifier $p = 3.4260e - 23$, AAM $p = 1.3123e - 29$, VAE $p = 4.5204e - 29$, VGG (PCA) $p = 1.6982e - 22$, PCA $p = 2.1103e - 31$, AE $p = 2.1467e - 32$, ICA $p = 1.5940e - 30$, two-sided Welsch's t-test). Circles, median alignment per model (β-VAE, n=51; VGG (raw), n=23; Classifier, n=71; VAE, Variational AutoEncoder[1], n=50; AE, AutoEncoder[2], n=50; VGG (PCA)[3], n=41; PCA, n=41; ICA, n=50; AAM, Active Appearance Model[4], n=21). Teal boxplot - random baseline with sparseness matched to the 51 β-VAE models. Boxplot center is median, box extends to 25th and 75th percentiles, whiskers extend to the most extreme data that are not considered outliers, outliers are plotted individually. Source data are provided as a Source Data file.

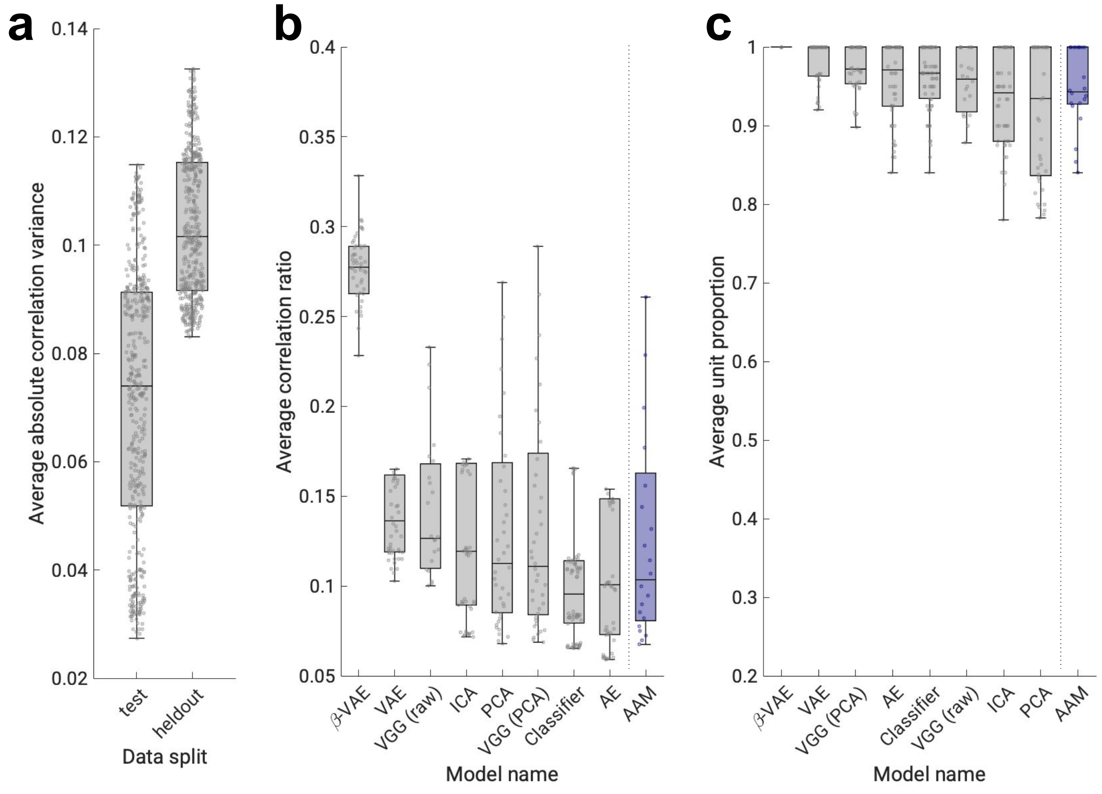

Supplementary Figure 2: **One-to-one correlation and diversity results broadly generalise to heldout faces.** **a.** Standard deviation of absolute Pearson correlation between the responses of single neurons and single model units when calculated using 2,100 test faces or 62 heldout faces. Each dot corresponds to a single model from across all model classes (n=449). Correlation scores are significantly more variable in the low-data regime of heldout faces ($p = 0.003$, two-sided Welsch's t-test). Boxplot center is median, box extends to 25th and 75th percentiles, whiskers extend to the most extreme data that are not considered outliers, outliers are plotted individually. Source data are provided as a Source Data file. **b.** Average correlation ratio scores calculated using heldout 62 face data. β-VAE scores are significantly higher than the other baselines (all $p < 0.01$, AAM $p = 3.3598e-09$, ICA $p = 3.6192e-11$, VGG (PCA) $p = 4.7524e-15$, PCA $p = 2.7462e-16$, VAE $p = 1.7377e-13$, AE $p = 9.5982e-15$, Classifier $p = 3.5077e-52$, VGG (raw) $p = 5.5346e-12$, two-sided Welsch's t-test). Circles, average correlation ratio score per model (β-VAE, n=51; VGG (raw), n=23; Classifier, n=71; VAE, Variational AutoEncoder[1], n=50; AE, AutoEncoder[2], n=50; VGG (PCA)[3], n=41; PCA, n=41; ICA, n=50; AAM, Active Appearance Model[4], n=21). Boxplot center is median, box extends to 25th and 75th percentiles, whiskers extend to the most extreme data that are not considered outliers, outliers are plotted individually. Source data are provided as a Source Data file. **c.** Average unit proportion scores calculated using heldout 62 face data. β-VAE scores are significantly higher than the other baselines apart from VAE and VGG (raw) (all $p < 0.01$, AAM $p = 0.0013$, ICA $p = 3.8819e-08$, VGG (PCA) $p = 0.0076$, PCA $p = 4.7032e-06$, VAE $p = 0.0788$, AE $p = 1.1799e-04$, Classifier $p = 1.2764e-05$, VGG (raw) $p = 0.0630$, two-sided Welsch's t-test). Circles, average unit proportion score per model (β-VAE, n=51; VGG (raw), n=23; Classifier, n=71; VAE, Variational AutoEncoder[1], n=50; AE, AutoEncoder[2], n=50; VGG (PCA)[3], n=41; PCA, n=41; ICA, n=50; AAM, Active Appearance Model[4], n=21). Boxplot center is median, box extends to 25th and 75th percentiles, whiskers extend to the most extreme data that are not considered outliers, outliers are plotted individually. Source data are provided as a Source Data file.

Supplementary Note: Variance explained

We calculated how much neural response variance was explained by the β-VAE and the baseline models (encoding variance explained), and how much variance in the model responses was explained by the neurons (decoding variance explained) (see Figure 7a-b). Both scores were calculated to get a better understanding of the overall profile of linearly accessible information overlap between the neural population and the model representations (see Figure 7c). β-VAE has slightly worse encoding performance compared to the AE and some other baselines is due to the nature of the disentangling objective. Disentangling in β-VAE models is achieved by increasing the weight of the compression term in the learning objective[5,6], which naturally discards information. The upshot of disentangling as shown in the paper, however, is that the representational form becomes more aligned with that of the neural population.

Note that single β-VAE units can account for more than 50% of the variance explained by all 50 units of the baseline with the highest encoding score (AAM) for around 10% of all neurons (see Figure 7d and Supplementary Figure 1). Given that a well disentangled β-VAE has approximately 15 good units, and 10% of the neural population also makes up around 15 neurons, this further supports our conclusion that β-VAE was able to discover a representation that is equivalent to a similarly sized subset of real neurons.

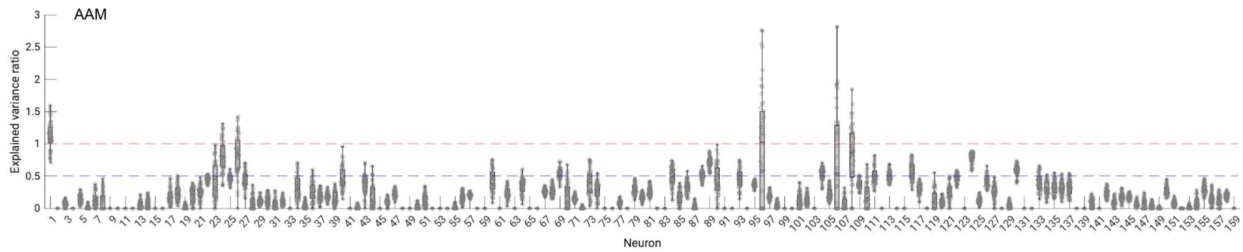

Supplementary Figure 3: **Per neuron variance explained by single β-VAE units compared to that explained by the full population of the best baseline (AAM).** Ratio of total variance explained for each neuron by the baseline model with the highest median encoding score shown in Figure 6a – AAM (50 units) – accounted for by single units from each of the 51 trained β-VAE models. Circles, neural explained variance using information from the best aligned β-VAE single unit. Points above red line - single β-VAE units account for more neural response variance than what is explained by 50 AAM units. Points above blue line - single β-VAE units account for more than 50% of neural response variance explained by 50 AAM units. Boxplot center is median, box extends to 25th and 75th percentiles, whiskers extend to the most extreme data that are not considered outliers, outliers are plotted individually. Source data are provided as a Source Data file.

Supplementary Note: Training β-VAE on a synthetic dataset of faces.

To demonstrate the general ability of β-VAE to recover a large set of disentangled semantically interpretable dimensions that span the space of faces, we trained it on a synthetic dataset of perfectly aligned faces from the "illumination" subset of the SUFR dataset[7]. The dataset contains 2,800 images of 400 identities generated using a 3D engine with 7 lighting conditions each. We used the same model architecture and training protocol as described in Methods to train β-VAE using images of 256x256 pixel resolution. The trained models discovered around 25 informative disentangled dimensions on average (which is more than double the number of dimensions discovered by well disentangled β-VAE models on the smaller and more noisy dataset of 2,100 faces used in the main results section). A visualisation of the learnt dimensions can be seen in "SUFR disentangling" file at `https://doi.org/10.6084/m9.figshare.c.5613197.v1`.

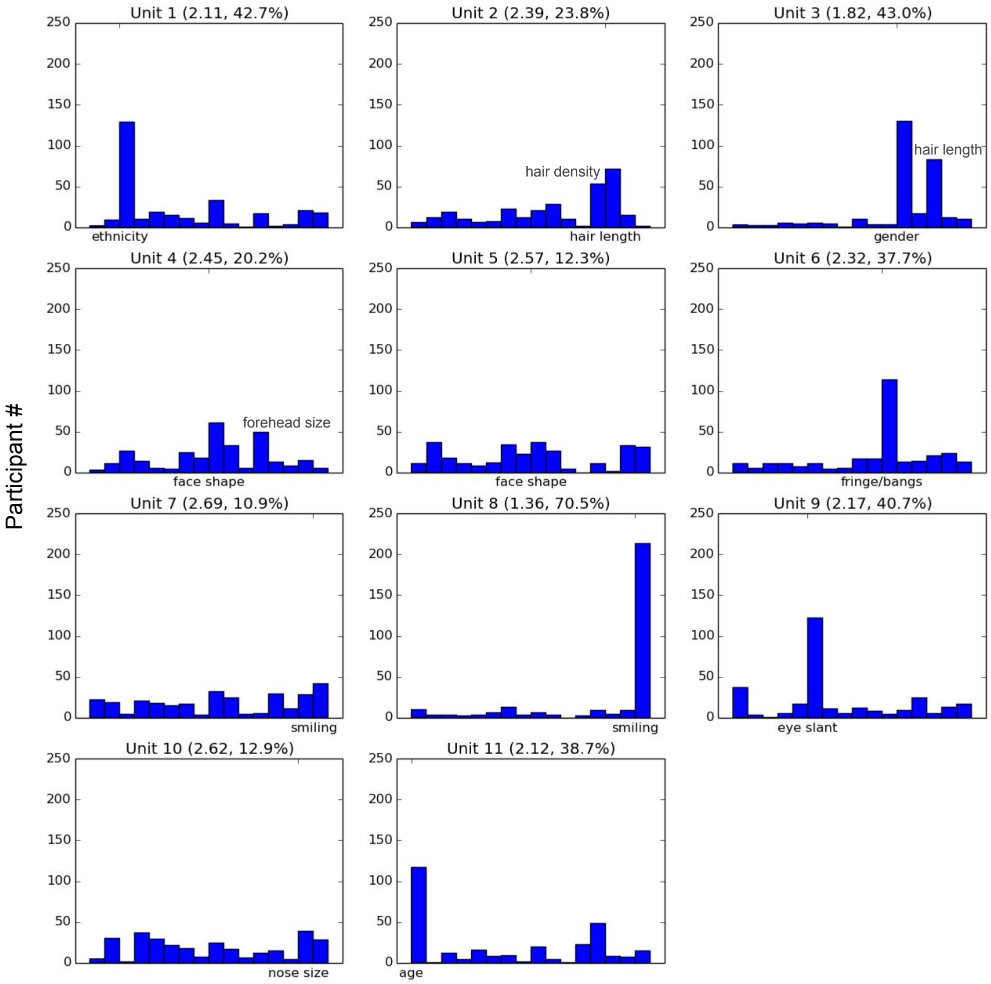

Supplementary Figure 4: **Latent traversal label agreement between human participants.** Number of participants choosing particular label options from 17 possibilities. Numbers in brackets indicate entropy in nats and maximum rater agreement as percentage of the 300 participants. Maximum entropy - 2.83. Source data are provided as a Source Data file.

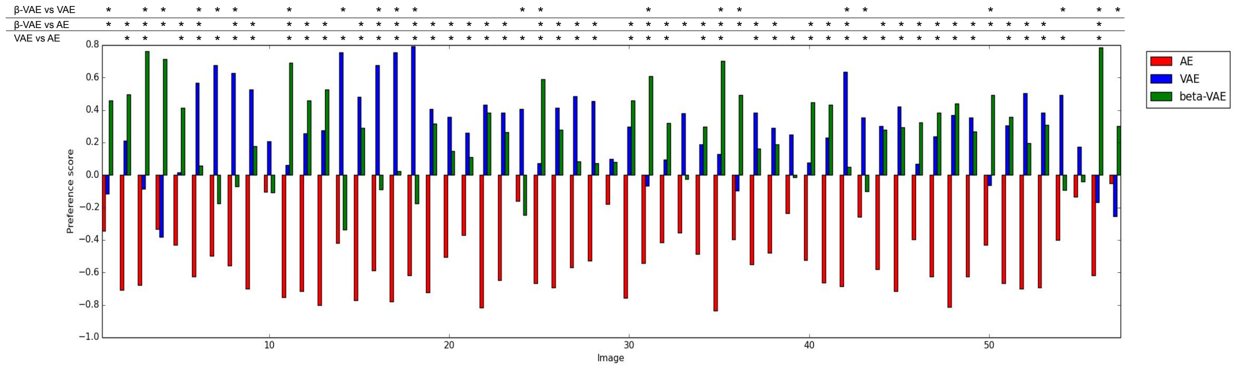

Supplementary Figure 5: **Subjective reconstruction quality breakdown per image.** Reconstructions by AE, VAE and β-VAE of novel 57 face images from 30, 27 and 12 neurons respectively were ranked by human participants in terms of their similarity to the original. “Best”: 1; “Ok”: 0; “Worst”: -1. Bars, average scores across 300 participants per image. Star, statistically significant difference according to Friedman post-hoc pairwise comparisons ($p < 0.05$). Source data are provided as a Source Data file.

Supplementary References

1. Kingma, D. P. & Welling, M. Auto-encoding variational bayes. *ICLR* (2014).

2. Hinton, G. E. & Salakhutdinov, R. Reducing the dimensionality of data with neural networks. *Science* **313**, 504–507 (2006).

3. Parkhi, O., Vedaldi, A., & Zisserman, A. Deep face recognition. *BMVC* (2015).

4. Chang, L. & Tsao, D. Y. The code for facial identity in the primate brain. *Cell* **169**, 1013–1028 (2017).

5. Higgins, I. *et al.* β-VAE: Learning basic visual concepts with a constrained variational framework. *ICLR* (2016).

6. Burgess, C. P. *et al.* Understanding disentangling in β-VAE. *arXiv preprint arXiv:1804.03599* (2018).

7. Leibo, J., Liao, Q. & Poggio, T. SUFR-W (Subtasks of unconstrained face recognition) (2019). URL `https://doi.org/10.7910/DVN/SJV7QK`.
