# Supplementary material for: Unsupervised deep learning identifies semantic disentanglement in single inferotemporal face patch neurons
Source: Nat Commun. 2021 Nov 9;12:6456. doi: 10.1038/s41467-021-26751-5 (PMC8578601; doi:10.1038/s41467-021-26751-5)
Supplement: Supplementary file 3 — Reporting summary. [file 41467_2021_26751_MOESM3_ESM.pdf]

nature research

Corresponding author(s): Irina Higgins

Last updated by author(s): Jun 21, 2021

Reporting Summary

Nature Research wishes to improve the reproducibility of the work that we publish. This form provides structure for consistency and transparency in reporting. For further information on Nature Research policies, see our Editorial Policies and the Editorial Policy Checklist.

Statistics

For all statistical analyses, confirm that the following items are present in the figure legend, table legend, main text, or Methods section.

n/a	Confirmed	
[ ]	[x]	The exact sample size (n) for each experimental group/condition, given as a discrete number and unit of measurement
[ ]	[x]	A statement on whether measurements were taken from distinct samples or whether the same sample was measured repeatedly
[ ]	[x]	The statistical test(s) used AND whether they are one- or two-sided<br>*Only common tests should be described solely by name; describe more complex techniques in the Methods section.*
[x]	[ ]	A description of all covariates tested
[x]	[ ]	A description of any assumptions or corrections, such as tests of normality and adjustment for multiple comparisons
[ ]	[x]	A full description of the statistical parameters including central tendency (e.g. means) or other basic estimates (e.g. regression coefficient) AND variation (e.g. standard deviation) or associated estimates of uncertainty (e.g. confidence intervals)
[ ]	[x]	For null hypothesis testing, the test statistic (e.g. F, t, r) with confidence intervals, effect sizes, degrees of freedom and P value noted<br>*Give P values as exact values whenever suitable.*
[x]	[ ]	For Bayesian analysis, information on the choice of priors and Markov chain Monte Carlo settings
[x]	[ ]	For hierarchical and complex designs, identification of the appropriate level for tests and full reporting of outcomes
[ ]	[x]	Estimates of effect sizes (e.g. Cohen's d, Pearson's r), indicating how they were calculated

Our web collection on statistics for biologists contains articles on many of the points above.

Software and code

Policy information about availability of computer code

Data collection	We used our own Python 2.7 implementation of the data pre-processing pipeline, and our own implementation of the beta-VAE model (as well as AE, VAE and Classifier models) in TensorFlow 1 (although publicly available implementations exist, e.g. https://github.com/google-research/disentanglement_lib). We used sklearn 0.2 PCA and fastICA implementations. For VGG-Face model we used a pre-trained MATLAB R2017b implementation from http://www.vlfeat.org/matconvnet/pretrained/.
Data analysis	We used our own MATLAB R2017b implementation of the UDR and alignment scores for data analysis (although a publicly available implementation exists in https://github.com/google-research/disentanglement_lib). We used MATLAB R2017b Lasso regression (lasso), Spearman correlation (corr), and Welsch's t-test (ttest2) implementations for analysis. We also used used Kuhn–Munkres algorithm MatLab implementation from https://www.mathworks.com/matlabcentral/fileexchange/62970-hungarian-algorithm-for-linear-sum-assignment-problem and Python 3.6 scipy stats.friedmanchisquare implementation of Friedman post-hoc pairwise comparisons test.

For manuscripts utilizing custom algorithms or software that are central to the research but not yet described in published literature, software must be made available to editors and reviewers. We strongly encourage code deposition in a community repository (e.g. GitHub). See the Nature Research guidelines for submitting code & software for further information.

Data

Policy information about availability of data

All manuscripts must include a data availability statement. This statement should provide the following information, where applicable:

- Accession codes, unique identifiers, or web links for publicly available datasets
- A list of figures that have associated raw data
- A description of any restrictions on data availability

The unprocessed responses of all models to the 2,162 face images generated in this study have been deposited in the figshare database under accession code TBC. The raw neural data supporting the current study are available under restricted access because of the complexity of the customized data structure and the size of the data; access can be obtained by contacting Le Chang (stevenlechang@gmail.com) or Doris Tsao (tsao.doris@gmail.com). The raw psychophysics data are protected and are not available due to data privacy laws. All the data generated in this study and presented in the figures are provided in the Supplementary Information/Source Data file. The face image data used in this study are available in the corresponding databases: FERET face database (https://www.nist.gov/itl/iad/image-group/color-feret-database), CVL face database (http://lrv.fri.uni-lj.si/facedb.html), MR2 face database (http://ninastrohminger.com/the-mr2), PEAL face database (http://www.jdl.ac.cn/peal/index.Html), AR face database (http://www2.ece.ohio-state.edu/~aleix/ARdatabase.html), Chicago face database (http://faculty.chicagobooth.edu/bernd.wittenbrink/cfd/download/download.html) and CelebA face database (http://mmlab.ie.cuhk.edu.hk/projects/CelebA.html). These databases do not allow further distribution of their images, so we cannot release the exact subset of images that were used in this study.

Field-specific reporting

Please select the one below that is the best fit for your research. If you are not sure, read the appropriate sections before making your selection.

☒ Life sciences ☐ Behavioural & social sciences ☐ Ecological, evolutionary & environmental sciences

For a reference copy of the document with all sections, see nature.com/documents/nr-reporting-summary-flat.pdf

Life sciences study design

All studies must disclose on these points even when the disclosure is negative.

Sample size	For each model class we trained a large number of model instances with different hyperparameters, which after filtering resulted in the average sample size of 44.6 (min 21, max 64), which is sufficient to achieve acceptable effect size of Cohen's d=0.8 at p=0.05 significance level. Where the model class did not admit hyperparameter variations, we created other variations (e.g. by randomly sub-sampling the units or taking different subsets of the top-K principal components of the model representation) to maximise the sample size. All details are reported in Online Methods
Data exclusions	We excluded model instances that failed to train or which failed to explain a threshold amount of response variance within the neural populations. All details are reported in Online Methods.
Replication	We trained 10 seeds of each model with the same hyperparameters where appropriate (see Online Methods) and found that the results broadly held regardless of the particular random initialisation used.
Randomization	This is not relevant to our study, because group allocation was based on model class, which we trained ourselves and it was not possible to randomize.
Blinding	Since we trained the models and wrote the script for analyzing the data ourselves, we were not blind to group allocations.

Reporting for specific materials, systems and methods

We require information from authors about some types of materials, experimental systems and methods used in many studies. Here, indicate whether each material, system or method listed is relevant to your study. If you are not sure if a list item applies to your research, read the appropriate section before selecting a response.

Materials & experimental systems

n/a	Involved in the study	
☒	☐	Antibodies
☒	☐	Eukaryotic cell lines
☒	☐	Palaeontology and archaeology
☐	☒	Animals and other organisms
☐	☒	Human research participants
☒	☐	Clinical data
☒	☐	Dual use research of concern

Methods

n/a	Involved in the study	
☒	☐	ChIP-seq
☒	☐	Flow cytometry
☒	☐	MRI-based neuroimaging

Animals and other organisms

Policy information about studies involving animals; ARRIVE guidelines recommended for reporting animal research

Laboratory animals	The data was collected from two male rhesus macaques (Macaca mulatta) of 7-10 years old, originally for another study (ref 6 in the paper).
Wild animals	This study did not involve wild animals
Field-collected samples	This study did not involve samples collected in the field
Ethics oversight	The data was collected by ref 6 in the manuscript under Caltech Animal Protocol Application Number: #1574-19

Note that full information on the approval of the study protocol must also be provided in the manuscript.

Human research participants

Policy information about studies involving human research participants

Population characteristics	600 human participants were recruited, 300 for each of the two studies, age 30.81 +- 11.07, 117 females for identifying transformations applied to faces, and age 30.75 +- 10.57, 123 females for comparing face reconstructions.
Recruitment	The participants were recruited through the Prolific crowd-sourcing platform, after reading and signing the informed consent form. The participants were paid for their time.
Ethics oversight	HuBREC, DeepMind

Note that full information on the approval of the study protocol must also be provided in the manuscript.
